# No significant differences in 60-day postoperative complication rates between conventional and shortened stems

**DOI:** 10.1186/s40634-023-00696-8

**Published:** 2023-12-28

**Authors:** Guillaume Girardot, Sylvain Guy, ReSurg, Sonia Ramos-Pascual, Sonia Dubreuil, Mo Saffarini, Nicolas Bonin

**Affiliations:** Lyon Ortho Clinic, Clinique de La Sauvegarde, Ramsay Santé, 29B Avenue Des Sources, Lyon, France

**Keywords:** THA, Total hip arthroplasty, Short stems, Total hip replacement, Radiographic outcomes

## Abstract

**Purpose:**

To compare 60-day complication rates, radiographic outcomes, and clinical outcomes following primary THA with conventional versus shortened stems, in a large cohort study.

**Methods:**

The authors reviewed a consecutive series of 800 primary THAs, of which 781 met the inclusion/exclusion criteria: 395 received a conventional stem and 386 received a shortened stem. Intraoperative and postoperative complications were noted. Radiographic and clinical assessments were performed preoperatively and 60 days after surgery.

**Results:**

Compared to conventional stems, shortened stems had significantly less intraoperative complications (2.8% vs 0.3%, *p* = 0.006), but no significant differences in complications that did not require reoperation (1.0% vs 1.3%, *p* = 0.620), complications that required reoperation without stem revision (2.0% vs 1.0%, *p* = 0.384), and complications that required stem revision (0.5% vs 0.5%, *p* = 1.000). Four hips (two from each group) required stem revision and were thus excluded from 60-day assessment. There were no significant differences between groups in subsidence ≥ 3 mm (1.0% vs 0.5%, *p* = 0.686), alignment (90.3%vs 86.7%, *p* = 0.192), net change in offset (within 3 mm, 32.3% vs 30.5%, *p* = 0.097), and limb length discrepancy (3.0 ± 2.6 mm vs 2.9 ± 2.4 mm, *p* = 0.695). Compared to conventional stems, shortened stems had significantly better preoperative mHHS (56.5 ± 18.5 vs 64.5 ± 13.5, *p* < 0.001), and significantly lower net improvement in mHHS (29.9 ± 17.1 vs 24.4 ± 15.0, *p* < 0.001), but no significant differences in postoperative mHHS (87.3 ± 11.9 vs 89.4 ± 9.6, *p* = 0.109).

**Conclusions:**

There were no significant differences between conventional and shortened stems in terms of postoperative complication rates, radiographic outcomes, and postoperative mHHS. However, patients implanted with shortened stems had less intraoperative complications, but lower net improvement in mHHS.

**Level of Evidence:**

Level IV, Retrospective comparative cohort study

**Supplementary Information:**

The online version contains supplementary material available at 10.1186/s40634-023-00696-8.

## Introduction

The length of cementless femoral stems for primary total hip arthroplasty (THA) has evolved over the last decades. In recent years, the use of short stems has increased, as they preserve diaphyseal bone stock for future revisions, decrease stress shielding at the proximal femur, and are more easily implanted through minimally invasive approaches [23, 44]. According to the cementless stem classification by Kheir et al. [17], short stems can be classified into four types, (i) femoral neck, (ii) calcar loading, (iii) calcar loading with lateral flare, and (iv) shortened tapered. Shortened tapered stems were developed as a compromise between short and conventional-length stems, as they provide metaphyseal anchorage, while facilitating axial alignment. This stem design has proven satisfactory clinical and radiographic outcomes and is being increasingly used for the general population [13, 17].

Five systematic reviews have directly compared conventional versus short stems [1, 12, 23, 43, 44], concluding that clinical outcomes and survival rates were similar. In contrast, radiographic outcomes were not widely evaluated; three systematic reviews reported contradictory findings regarding bone mineral density [23, 43, 44], while one systematic review reported no significant differences in femoral offset and limb length discrepancy [12]. A number of comparative clinical studies have reported radiographic outcomes of conventional versus short stems, with cohorts varying between 25–132 per group [13, 16, 19–22, 36, 37, 42], which may be underpowered to detect significant differences across groups, considering the small incidence of subsidence (0–2%) and misalignment (0–5%) [13, 16, 19, 22, 36]. In terms of subsidence, Kato et al. [16] reported only one case ≥ 2 mm in the conventional group and Shin et al. [36] reported only one case ≥ 2 mm in the short group, while Lacko et al. [22] and Kim et al. [19] reported no cases ≥ 2 mm and ≥ 3 mm respectively in either group. In terms of misalignment, Kim et al. [19] reported no cases ≥ 5° in either group, while Shin et al. [36] reported 2 versus 1 case ≥ 5° in the short and conventional groups respectively (*p* = 0.554).

To the authors’ knowledge, there are no published studies that compare early radiographic outcomes of large cohorts of conventional versus shortened stems, particularly subsidence and misalignment. The authors of the present study were interested in evaluating if a shortened stem could be used in the general population requiring primary THA without increasing the rates of early complications. Therefore, the purpose of the present study was to compare 60-day complication rates, radiographic outcomes, and clinical outcomes following primary THA with conventional versus shortened stems, in a large cohort study. The null hypothesis was that there would be no differences in any outcomes across groups.

## Materials and methods

The authors reviewed the records of a consecutive series of 800 primary THAs, performed by the same experienced surgeon (NB) between February 2013 and July 2017, who systematically used the direct anterior (Hueter) approach for all primary THAs. Prior to February 2013, the surgeon had performed over 450 primary THAs using the direct anterior approach. From 2013 to 2014 the surgeon exclusively used a conventional-length collared stem (Hype, Serf, Décines-Charpieu, France), while from 2015 to 2017 the surgeon transitioned to a shortened collared stem (Symbol, Dedienne Santé, Mauguio, France). At the beginning of the transition period, the surgeon only had one instrumentation set available for the shortened stem, and implanted the shortened stem in the first patient he operated each day. Progressively, a greater number of instrumentation sets were available on the day of surgery, until he was able to exclusively use the shortened stem. During the transition period, the surgeon implanted 205 conventional stems and 386 shortened stems. The conventional collared stem is a Corail-like straight stem manufactured from titanium alloy and fully coated, first with unalloyed titanium and then hydroxyapatite; it is classified as a type 2 using Kheir’s classification for cementless femoral stems [17]. The shortened collared stem is manufactured from titanium alloy and fully coated with hydroxyapatite; it is classified as a type 1D using Kheir’s classification for cementless femoral stems [17]. The surgeon transitioned from a conventional straight stem to a shortened metaphyseal-filling stem because it permitted a more minimally-invasive and bone-sparing surgery.

**Fig. 1:**
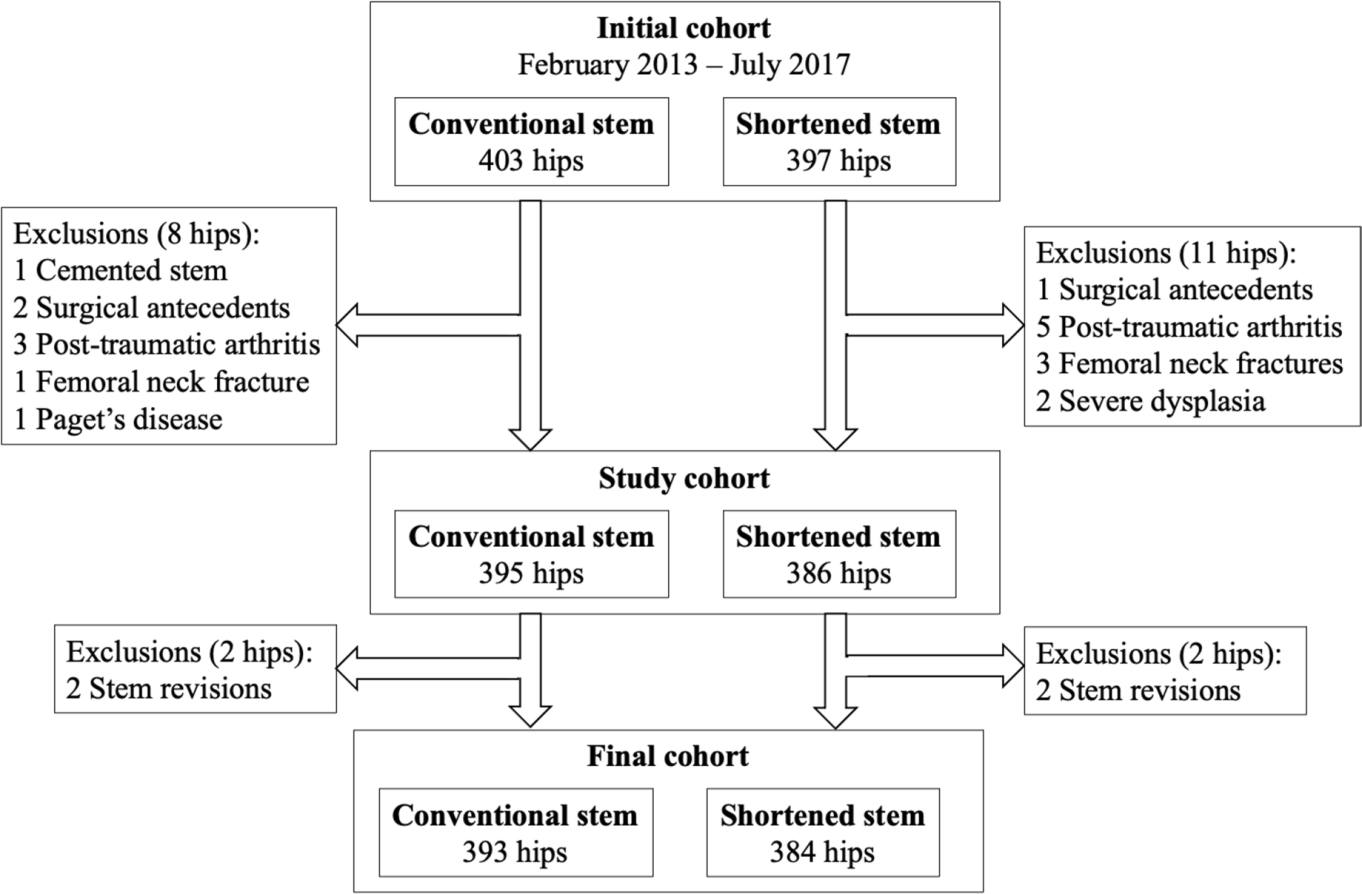
Flowchart describing the initial cohort, study cohort, and final cohort

Patients were excluded from the study if they had (i) surgical antecedents in the ipsilateral hip other than soft-tissue repairs/releases (*n*=3), (ii) cemented stem fixation (*n* = 1), or (iii) any of the following surgical indications: femoral neck fracture (*n* = 4), severe (Crowe III and IV) dysplasia (*n* = 2), Paget’s disease (*n* = 1), and post-traumatic arthritis (*n* = 8) (Fig. 1). This left a final cohort of 781 hips, of which 395 received the conventional stem and 386 received the shortened stem. The two groups had similar age (*p* = 0.879), BMI (*p* = 0.904), and sex distribution (*p* = 0.720), but significantly different surgical indications (*p* = 0.007) and Charnley comorbidity classes (*p* < 0.001), with the shortened stem group having a greater proportion of class C patients (28.9% vs 37.6%) (Table 1). This study was approved by the institutional review board of ‘GCS Ramsay Santé pour l’Enseignement et la Recherche’ (COS-RGDS-2023–01-002-BONIN-N). Informed consent was obtained from all individual participants included in the study.

**Table 1:** Patient demographics, and preoperative clinical scores and radiographic measurements stratified by stem type

	Conventional stem *n* = 395		Shortened stem *n* = 386		*p*-value
	Mean ± SD	(Range)	Mean ± SD	(Range)	
	*n* (%)		*n* (%)		
**Age**	64.6 ± 13.6	(19.2–92.5)	64.6 ± 13.2	(19.8–94.9)	0.879
**Body mass index (BMI)**	26.2 ± 4.8	(15.2–56.8)	26.1 ± 4.3	(16.4–44.4)	0.904
**Sex**					0.720
Female	203 (51.4%)		192 (49.7%)		
Male	192 (48.6%)		194 (50.3%)		
**Charnley comorbidity classification**					< 0.001
A	240 (60.8%)		184 (47.7%)		
B	136 (34.4%)		135 (35.0%)		
C	19 (4.8%)		67 (17.4%)		
**Surgical indication**					0.007
Avascular necrosis	24 (6.1%)		14 (3.6%)		
Primary OA	304 (77.0%)		333 (86.3%)		
Rapidly destructive OA	22 (5.6%)		12 (3.1%)		
Rheumatoid arthritis	3 (0.8%)		3 (0.8%)		
Secondary OA due to acetabular protrusio	17 (4.3%)		4 (1.0%)		
Secondary OA due to hip dysplasia	25 (6.3%)		20 (5.2%)		
**Modified Harris hip score**	56.5 ± 18.5	(12–95)	64.5 ± 13.5	(14–90)	< 0.001
**Canal calcar ratio (CCR)**	0.48 ± 0.10	(0.28–0.84)	0.48 ± 0.09	(0.28–0.76)	0.984
**Cortical thickness index (CTI)**	0.55 ± 0.09	(0.12–0.75)	0.58 ± 0.08	(0.27–0.78)	< 0.001
**Dorr classification**					0.007
A	114 (28.9%)		145 (37.6%)		
B	241 (61.0%)		192 (49.7%)		
C	40 (10.1%)		49 (12.7%)		
**Femoral offset**	47.1 ± 9.3	(19.4–92.0)	48.6 ± 10.6	(26.3–108.0)	0.137
**Limb length discrepancy (LLD)**	-1.5 ± 4.8	(-28.1–16.6)	-2.21 ± 5.1	(-45.4–8.9)	0.115

*Abbreviations*: *SD* standard deviation, *OA* osteoarthritis

### Assessment of complications

Intraoperative complications were noted during surgery. In addition, the following were recorded throughout the first 60 days after surgery: complications that did not require reoperation (general and hip-related), complications that required reoperation without stem revision, and complications that required stem revision.

### Radiographic assessment

**Fig. 2:**
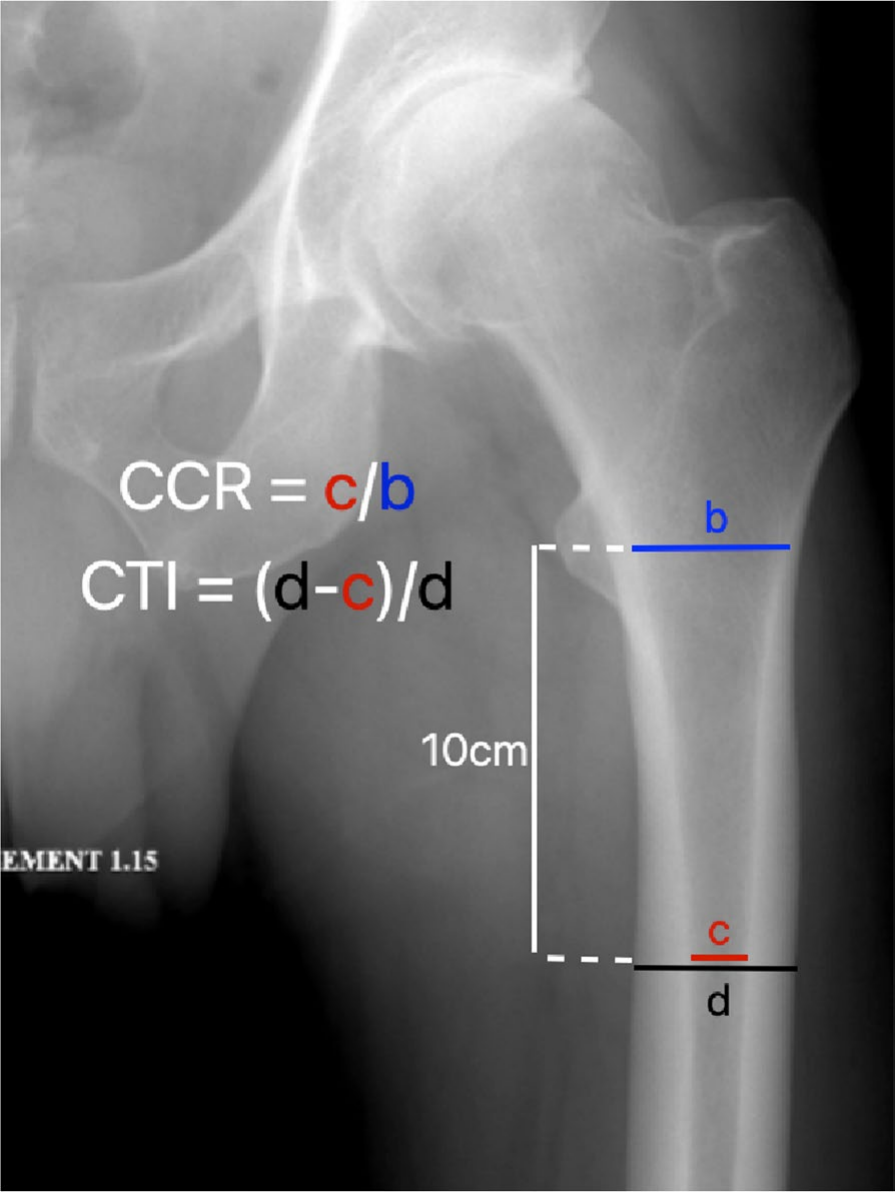
Cortical thickness index (CTI) and canal calcar ratio (CCR) were performed on preoperative anteroposterior pelvic radiographs, with CTI = (d – c) / d and CCR = c / b

Radiographic measurements were performed by either a resident orthopaedic surgeon (4 years experience) or a junior orthopaedic surgeon (5 years experience), both of whom assessed the same 20 radiographs to calculate interobserver agreement. Preoperative anteroposterior (AP) pelvic radiographs were assessed to evaluate femoral offset and limb length discrepancy (LLD), as well as femoral morphology according to Dorr classification [8], cortical thickness index (CTI) [29], and canal calcar ratio (CCR) [7] (Fig. 2).

**Fig. 3:**
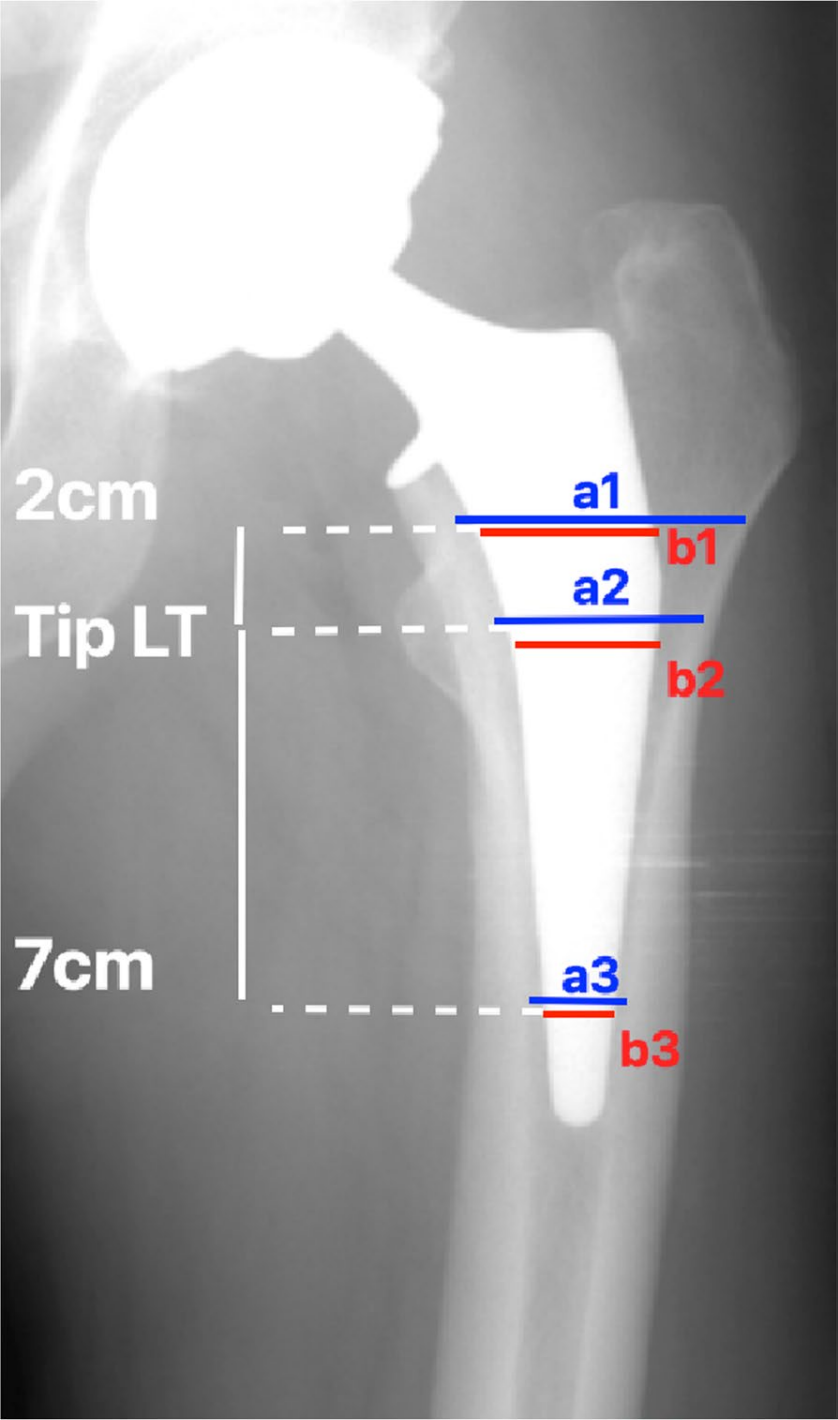
Canal fill ratio (CFR) was calculated by dividing the femoral stem width by the endosteal diameter width at 3 levels, with the lesser trochanter (LT) as reference point: (i) 2 cm above the tip of the LT, (ii) at the level of the tip of the LT, and (iii) 7 cm below the tip of the LT

Postoperative 60-day AP pelvic and lateral hip radiographs were assessed to evaluate femoral offset [3], LLD, stem alignment (varus/valgus if stem axis ≥ 3° from neutral). Stem subsidence was measured from the tip of the greater trochanter to the shoulder of the stem, and taken as a difference of ≥ 3 mm between immediate and 60-day AP pelvic radiographs [3]. The canal fill ratio (CFR) was calculated by dividing the femoral stem width by the endosteal diameter width at 3 levels, with the lesser trochanter (LT) as reference point: (i) 2 cm above the tip of the LT, (ii) at the level of the tip of the LT, and (iii) 7 cm below the tip of the LT [6] (Fig. 3).

### Clinical assessment

Patients were evaluated preoperatively and at 60 days after surgery using the modified Harris hip score (mHHS).

### Statistical analysis

An a priori sample size calculation indicated that 394 patients per group were needed to determine the significance of 2% difference in incidence of subsidence and misalignment between conventional and shortened stems [36], with a statistical power of 80%. Descriptive statistics were used to summarise demographic data, clinical scores and radiographic measurements. A cohort-specific minimal clinically important difference (MCID) was calculated as half of the standard deviation of the net change of the mHHS; which was 8.9 points for the conventional stems and 7.4 points for the shortened stems. For categorical variables, comparisons between groups were performed using Fisher’s tests or Chi-squared tests, respectively for binary and non-binary variables. Normality of continuous variables was assessed through Shapiro–Wilk tests. For continuous variables, comparisons between groups were performed using student’s t-tests or Wilcoxon signed rank tests, respectively for normally distributed and non-normally distributed variables. Since the two groups had significantly different surgical indications and Charnley comorbidity classes, linear regression analyses were performed to account for the effect of these differences on postoperative mHHS, as well as to determine possible associations of postoperative mHHS with the independent variables (sex, age, BMI, Charnley comorbidity classification, surgical indication, stem implanted, CCR, CTI, Dorr classification, preoperative femoral offset, and preoperative LLD); associations were presented as regression estimates (β) with their corresponding 95% confidence intervals (CI) and *p*-values. Multivariable linear regression analyses were performed after selection of pertinent variables using directed acyclic graphs (DAG) [38] (Additional file 1: Appendix 1). Interobserver agreement was assessed for all radiographic measurements using Gwet’s AC [11] or intraclass correlation coefficients (ICC), respectively for categorical and continuous variables, and these were interpreted as follows: < 0.40 poor; 0.40–0.59 fair; 0.60–0.74 good, and > 0.75 excellent [4]. Interobserver agreement was excellent or good for all radiographic measurements (Table 2). Statistical analyses were conducted using R, version 4.1.3 (R Foundation for Statistical Computing, Vienna, Austria). *P*-values < 0.05 were considered statistically significant.

**Table 2:** Interobserver agreement for radiographic measurements

	Inter-observer agreement			
	Coefficient	95% CI		*p*-value
**Preoperative canal calcar ratio (CCR)**	0.63	(0.26	–0.84)	< *0.001*
**Preoperative cortical thickness index (CTI)**	0.78	(0.54	–0.91)	< *0.001*
**Preoperative Dorr**	0.79	(0.56	–1.00)	< *0.001*
**Femoral offset**				
Preoperative	0.96	(0.90	–0.98)	< *0.001*
Postoperative	0.84	(0.59	–0.94)	< *0.001*
**Limb length discrepancy (LLD)**				
Preoperative	0.69	(0.37	–0.86)	< *0.001*
Postoperative	0.69	(0.37	–0.86)	< *0.001*
**Postoperative subsidence ≥ 3 mm**	0.69	(0.37	–0.86)	< *0.001*
**Postoperative alignment**	1.00	(1.00	–1.00)	< *0.001*
**Postoperative canal fill ratio (CFR)**				
2 cm above the lesser trochanter	0.61	(0.14	–0.84)	< *0.001*
at the level of the lesser trochanter	0.72	(0.42	–0.88)	< *0.001*
7 cm below the lesser trochanter	0.73	(0.44	–0.88)	< *0.001*

*Abbreviations*: *CI* confidence intervals

Cicchetti gives the following often quoted guidelines for interpretation of agreement measures: < 0.40 poor; 0.40–0.59 fair; 0.60–0.74 good, 0.75–1.00 excellent

## Results

### Complications, reoperations, and stem revisions

Of the 395 hips implanted with conventional stems, 11 had intraoperative complications, 1 had a general complication that did not require reoperation, 3 had hip-related complications that did not require reoperation, 8 had complications that required reoperation without stem revision, and 2 had complications that required stem revision (Table 3). Of the 386 hips implanted with shortened stems, 1 had an intraoperative complication, 2 had general complications that did not require reoperation, 3 had hip-related complications that did not require reoperation, 4 had complications that required reoperation without stem revision, and 2 had complications that required stem revision.

**Table 3:** Complications, reoperations, and stem revisions stratified by stem type

Conventional stem *n* = 395		Shortened stem *n* = 386		*p*-value
	**n (%)**		**n (%)**	
**Intraoperative complications**	**11 (2.78%)**	**Intraoperative complications**	**1 (0.26%)**	**0.006**
*Stable calcar crack, left untreated*		*Stable calcar crack, left untreated*		
*Stable calcar crack, left untreated*				
*Stable calcar crack, left untreated*				
*Stable calcar crack, left untreated*				
*Stable calcar crack, left untreated*				
*Unstable calcar crack, treated with cerclage*				
*Unstable calcar crack, treated with cerclage*				
*Stable greater trochanter crack, left untreated*				
*Stable greater trochanter crack, left untreated*				
*Stable greater trochanter crack, left untreated*				
*Unstable greater trochanter crack, treated with cerclage*				
**60-day general complications that did not require reoperation**	**1 (0.25%)**	**60-day general complications that did not require reoperation**	**2 (0.52%)**	**0.620**
*Skin rash (erysipelas) at 60 days PO, treated with antibiotics*		*Pulmonary embolism at 11 days PO, treated with anticoagulants*		
		*Stroke at 8 days PO, treated with anticoagulants*		
**60-day hip-related complications that did not require reoperation**	**3 (0.76%)**	**60-day hip-related complications that did not require reoperation**	**3 (0.78%)**	**1.000**
*Crack in the proximal femur at 60 days PO, supervised but left untreated*		*Fracture of the greater trochanter at 53 days PO, supervised but left untreated*		
*Crack in the proximal femur at 50 days PO, supervised but left untreated*		*Femoral crack at 18 days PO, supervised but left untreated*		
*Undisplaced fracture of the greater trochanter due to a fall at 13 days PO, supervised but left untreated*		*Femoral crack due to a fall at 14 days PO, supervised but left untreated*		
**60-day complications that required reoperation without stem revision**	**8 (2.03%)**	**60-day complications that required reoperation without stem revision**	**4 (1.04%)**	**0.384**
*Cup migration at 57 days PO, treated with cup revision*		*Early infection at 8 days PO, required lavage and change of modular components*		
*Early infection at 21 days PO, required lavage and change of modular components*		*Early superficial infection at 18 days PO, required superficial lavage*		
*Early infection at 15 days PO, required lavage and change of modular components*		*Femoral fracture due to a fall at 17 days PO, treated with osteosynthesis*		
*Early infection at 50 days PO, required lavage and change of modular components*		*Femoral fracture due to a fall at 13 days PO, treated with osteosynthesis*		
*Early superficial infection at 25 days PO, required superficial lavage*				
*Femoral fracture due to a fall at 8 days PO, treated with osteosynthesis*				
*Intraoperative unstable trochanter crack, treated with cerclage during surgery, but resulted in change in stem position after surgery, thus required osteosynthesis and plate fixation 5 days after surgery*				
*Skin burn during surgery, required skin graft 12 days after surgery*				
**60-day complications that required stem revision**	**2 (0.51%)**	**60-day complications that required stem revision**	**2 (0.52%)**	**1.000**
*Early infection at 39 days PO, required stem and cup revision*		*Early infection at 13 days PO, required stem and cup revision*		
*Femoral fracture due to a fall at 8 days PO, required stem and cup revision and osteosynthesis*		*Femoral fracture due to a fall at 5 days PO, required stem revision and osteosynthesis*		

*Abbreviations*: *PO* postoperative

Compared to hips implanted with conventional stems, those implanted with shortened stems had a significantly lower rate of intraoperative complications (2.8% vs 0.3%, *p* = 0.006), but there were no significant differences in rates of general complications that did not require reoperation (0.3% vs 0.5%, *p* = 0.620), hip-related complications that did not require reoperation (0.8% vs 0.8%, *p* = 1.000), complications that required reoperation without stem revision (2.0% vs 1.0%, *p* = 0.384), and complications that required stem revision (0.5% vs 0.5%, *p* = 1.000).

It is important to note that the 4 hips that underwent stem revision were excluded from radiographic and clinical assessments, which enabled evaluation at 60 days for 393 hips implanted with conventional stems and 384 hips implanted with shortened stems.

### Radiographic measurements

Compared to hips implanted with conventional stems, those implanted with shortened stems had significantly higher CFR at the level of the lesser trochanter (0.68 ± 0.13 vs 0.76 ± 0.13, *p* < 0.001) and 7 cm below the lesser trochanter (0.93 ± 0.16 vs 0.96 ± 0.16, *p* = 0.002), but there were no significant differences in CFR 2 cm above the lesser trochanter (0.64 ± 0.14 vs 0.66 ± 0.16, *p* = 0.059) (Table 4). Furthermore, there were no significant differences between the two groups in terms of subsidence ≥ 3 mm (1.0% vs 0.5%, *p* = 0.686), alignment (aligned, 90.3%vs 86.7%, *p* = 0.192), net change in offset (within 3 mm, 32.3% vs 30.5%, *p* = 0.097), and LLD (3.0 ± 2.6 mm vs 2.9 ± 2.4 mm, *p* = 0.695).

**Table 4:** Clinical and radiographic outcomes stratified by stem type

	Conventional stem *n* = 393		Shortened stem *n* = 384		*p*-value
	Mean ± SD	(Range)	Mean ± SD	(Range)	
	n (%)		n (%)		
**Modified Harris hip score**					
Postoperative	87.3 ± 11.9	(47–100)	89.3 ± 9.6	(56–100)	0.109
Net improvement	29.9 ± 17.7	(-27–80)	24.3 ± 14.8	(-21–68)	< 0.001
Minimal clinically important difference	330 (84.0%)		314 (81.8%)		0.473
**Canal fill ratio (CFR)**					
2 cm above the lesser trochanter	0.64 ± 0.14	(0.42–1.06)	0.66 ± 0.16	(0.34–1.35)	0.059
At the level of the lesser trochanter	0.68 ± 0.13	(0.46–1.07)	0.76 ± 0.13	(0.16–1.12)	< 0.001
7 cm below the lesser trochanter	0.93 ± 0.16	(0.55–1.52)	0.96 ± 0.16	(0.54–1.47)	0.002
**Femoral offset**					
Postoperative	47.9 ± 9.5	(10.3–98.0)	49.8 ± 8.6	(28.8–105.0)	< 0.001
Absolute net change (continuous)	6.5 ± 7.2	(0.0–46.0)	6.4 ± 5.9	(0.0–43.0)	0.327
Net change (categorical)					0.097
Offset increased by ≥ 3 mm	140 (35.6%)		163 (42.4%)		
Offset remained within 3 mm	127 (32.3%)		117 (30.5%)		
Offset decreased by ≥ 3 mm	118 (30.0%)		94 (24.5%)		
Missing	8 (2.0%)		10 (2.6%)		
**Limb length discrepancy (LLD)**					
Absolute difference	3.0 ± 2.6	(0.0–25.3)	2.9 ± 2.4	(0.0–22.2)	0.695
Difference (categorical)					0.821
Difference ≥ 3 mm	144 (36.6%)		146 (38.0%)		
Difference within 3 mm	227 (57.8%)		221 (57.6%)		
Missing	22 (5.6%)		17 (4.4%)		
**Subsidence ≥ 3 mm**	4 (1.0%)		2 (0.5%)		0.686
**Alignment**					0.192
Aligned within 3°	355 (90.3%)		333 (86.7%)		
Valgus	10 (2.5%)		15 (3.9%)		
Varus	23 (5.9%)		33 (8.6%)		
Missing	5 (1.3%)		3 (0.8%)		

*Abbreviations*: *SD* standard deviation

### Clinical scores

Compared to hips implanted with conventional stems, those implanted with shortened stems had significantly better preoperative mHHS (56.5 ± 18.5 vs 64.5 ± 13.5, *p* < 0.001), and significantly lower net improvement in mHHS (29.9 ± 17.1 vs 24.4 ± 15.0, *p* < 0.001), but there were no significant differences in postoperative mHHS (87.3 ± 11.9 vs 89.4 ± 9.6, *p* = 0.109), nor in the proportion of patients that achieved the cohort-specific MCID in mHHS (84.0% vs 81.8%, *p* = 0.473) (Table 4). Moreover, there were no significant differences in mHHS when stratifying patients according to their change in offset (Table 5). Univariable linear regression analyses revealed that postoperative mHHS decreased with age (β = -0.1; 95%CI = -0.2– -0.1; *p* < 0.001) and BMI (β = -0.5; 95%CI = -0.7– -0.4; *p* < 0.001), but increased with preoperative femoral offset (β = 0.1; 95%CI = 0.0–0.2; *p* = 0.022), and was greater for the male sex (β = 3.0; 95%CI = 1.5–4.6; *p* < 0.001) and for patients implanted with the shortened stem (β = 2.1; 95%CI = 0.5–3.6; *p* = 0.010); furthermore, multivariable analyses confirmed these associations (Table 6).

**Table 5:** Postoperative mHHS stratified by change in offset

	Conventional stem	Shortened stem
	Mean ± SD	Mean ± SD
Offset increased by ≥ 3 mm	87.9 ± 11.9	89.6 ± 9.2
Offset remained within 3 mm	87.5 ± 11.6	89.0 ± 10.1
Offset decreased by ≥ 3 mm	86.4 ± 12.5	89.2 ± 9.8
*p*-value	0.608	0.860

*Abbreviations*: *SD* standard deviation

**Table 6:** Linear regression analyses for associations of variables with postoperative modified Harris Hip Score

	Univariable				Multivariable			
	*β*^a^	(95% CI)		*p*-value	*β*^a^	(95% CI)		*p*-*value*
**Age**	-0.1	(-0.2	– -0.1)	< 0.001	-0.1	(-0.2	– -0.1)	< 0.001
**Body mass index (BMI)**	-0.5	(-0.7	– -0.4)	< 0.001	-0.5	(-0.7	– -0.4)	< 0.001
**Sex: male**	3.0	(1.5	– 4.6)	< 0.001	3.0	(1.5	– 4.6)	< 0.001
**Charnley comorbidity classification**								
A	REF				REF			
B	-0.5	(-2.2	– 1.2)	0.585	-0.4	(-2.1	–1.3)	0.649
C	-2.0	(-4.7	– 0.7)	0.145	-1.4	(-4.1	–1.3)	0.308
**Surgical indication**								
Avascular necrosis	-0.4	(-4.2	– 3.3)	0.826	-0.4	(-4.2	– 3.3)	0.826
Primary OA	REF				REF			
Rapidly destructive OA	-3.7	(-7.5	– 0.1)	0.056	-3.7	(-7.5	– 0.1)	0.056
Rheumatoid arthritis^a^								
Secondary OA due to acetabular protrusio	-1.8	(-6.9	– 3.3)	0.484	-1.8	(-6.9	– 3.3)	0.484
Secondary OA due to hip dysplasia	-1.9	(-5.3	– 1.6)	0.287	-1.9	(-5.3	– 1.6)	0.287
**Stem implanted: shortened**	2.1	(0.5	– 3.6)	0.010	2.1	(0.5	– 3.6)	0.010
**Preoperative canal calcar ratio (CCR)**	-6.4	(-15.0	– 2.3)	0.148	-3.5	(-15.6	–8.7)	0.574
**Preoperative cortical thickness index (CTI)**	7.8	(-1.9	– 17.6)	0.114	4.5	(-9.6	–18.7)	0.529
**Preoperative Dorr classification**								
A	REF				REF			
B	-0.8	(-2.5	– 1.0)	0.382	-0.7	(-2.5	–1.2)	0.463
C	-0.3	(-3.1	– 2.4)	0.808	-0.3	(-3.2	–2.7)	0.867
**Preoperative femoral offset**	0.1	(0.0	– 0.2)	0.022	0.1	(0.0	– 0.2)	0.022
**Preoperative limb length discrepancy (LLD)**	0.1	(-0.1	– 0.2)	0.438	0.1	(-0.1	– 0.2)	0.438

*Abbreviations*: *SD* standard deviation, *CI* confidence interval, *OA* osteoarthritis

^a^Category excluded from analysis as it comprises less than 10 patients

## Discussion

The present study revealed similar 60-day outcomes in hips implanted with conventional and shortened stems, with no significant differences in rates of postoperative complications, radiographic outcomes, postoperative mHHS, or proportion of patients that achieved MCID in mHHS. It is worth noting, however, that patients implanted with shortened stems had 10 times less intraoperative complications (2.8% vs 0.26%, *p* = 0.006), higher CFR at the level of the lesser trochanter (0.68 ± 0.13 vs 0.76 ± 0.13, *p* < 0.001) and at 7 cm below the level of the lesser trochanter (0.93 ± 0.16 vs 0.96 ± 0.16, *p* = 0.002), as well as 5 points less net improvement in mHHS (29.9 ± 17.1 vs 24.4 ± 15.0, *p* < 0.001). Furthermore, regression analyses revealed that patients implanted with shortened stems had better postoperative mHHS. The present findings therefore partially refute the null hypothesis that there would be no differences in outcomes between conventional and shortened stems.

All intraoperative complications recorded were either femoral calcar cracks or greater trochanter cracks; one unstable trochanter crack was treated intraoperatively with cerclage, but resulted in change in stem position after surgery, thus required osteosynthesis and plate fixation 5 days after surgery; the remining intraoperative cracks required no postoperative treatment and had healed 60 days after surgery. The higher incidence of these cracks in hips implanted with conventional stems may be due to differences in stem design. The shortened stem is metaphyseal-filling and has a more curved shoulder than the conventional stem, thus may result in a smaller force against the calcar and the greater trochanter; furthermore, the shorter length requires a smaller femoral exposure, which is easier to achieve with the direct anterior approach. It is worth noting that shortened stems are more easily extracted than conventional stems during revision THA, and preserve diaphyseal bone stock for future revisions [23, 44].

The series of the present study represents a period during which the surgeon switched from conventional to shortened stems. The findings of the study suggest that the learning curve for this shortened stem was brief, as rates of complications, subsidence, misalignment, and LLD were similar or better for shortened stems compared to conventional stems.

Both groups had satisfactory clinical scores, with no significant differences in postoperative mHHS (87.3 ± 11.9 vs 89.3 ± 9.6, *p* = 0.109) or in the proportion of patients that achieved MCID in mHHS (84.0% vs 81.8%, *p* = 0.473); however, multivariable linear regression analyses revealed that postoperative mHHS was greater for patients implanted with the shortened stem (β = 2.1; 95%CI = 0.5–3.6; *p* = 0.010). Regression analyses also revealed that postoperative mHHS decreased with age (β = -0.1; 95%CI = -0.2– -0.1; *p* < 0.001) and BMI (β = -0.5; 95%CI = -0.7– -0.4; *p* < 0.001), but increased with preoperative femoral offset (β = 0.1; 95%CI = 0.0–0.2; *p* = 0.022), and was greater for the male sex (β = 3.0; 95%CI = 1.5–4.6; *p* < 0.001). The shortened stem group tended to have a greater proportion of males (48.6% vs 50.3%, *p* = 0.720) and greater femoral offset (47.1 ± 9.3 vs 48.6 ± 10.6, *p* = 0.137), which could have contributed to the 2-point higher postoperative mHHS of this group. Compared to patients implanted with conventional stems, those implanted with shortened stems had significantly higher preoperative mHHS, but significantly lower net improvement in mHHS, which could be explained by the ceiling effect of HHS [40]. Mean postoperative mHHS values in the present study (87.3 ± 11.9 vs 89.4 ± 9.6) are comparable to those reported in the literature for primary THA using other conventional or short cementless stems, which ranged between 82–97 points [10, 14, 18, 24, 34], with the study by Risitano et al. [34] also reporting a tendency for short stems to have better postoperative HHS than conventional stems (83 ± 13.4 vs 87 ± 14.1, *p* = 0.148).

A recent study on the Dutch Arthroplasty Register [39] analysed 228,917 cementless conventional stems and 3,352 cementless short stems and found no significant differences in 10-year stem revision rates (2.3% vs 3.0%), although today’s predominant short stems (Fitmore and Optimys) had lower revision rates than other less frequently used short stems (4.5%). In addition, prior clinical studies comparing conventional versus short stems found no significant differences in subsidence (0% vs 0% [19, 22]; 1% vs 0% [16]; 0% vs 2%, *p* = 0.554 [36]) or misalignment (0% vs 0% [19]; 2% vs 4%, *p* = 0.313 [36]; 1% vs 5%, *p* = 0.111 [13]); however, these clinical studies were underpowered to detect significant differences across groups. Based on this data, the present study performed an a priori sample size calculation to determine the number of patients that would be needed in each group to provide a significant difference in subsidence and misalignment. With a cohort of 403 conventional stems versus 397 shortened stems, the present study also found no significant differences in subsidence (1.0% vs 0.5%, *p* = 0.686) or misalignment (8.4% vs 12.5%, *p* = 0.192). These findings confirm the benefits of the shortened tapered stem design, which was developed as a compromise between short and conventional stems, to provide metaphyseal anchorage while facilitating axial alignment. Interestingly, two recent studies [28, 32] on short stems found a higher risk of subsidence in males and heavy-weight patients, with Mittelstaedt et al. [28] also reporting a higher risk of subsidence in patients aged < 65. However, it is important to note that these two studies implanted collarless stems.

Although both collared and collarless cementless stems provide excellent outcomes for primary THA, most recent clinical and biomechanical studies that compared collared versus collarless stems demonstrated that the collar could reduce subsidence [31, 33, 41], complications [5, 30], and radiolucent lines or pedestals [15, 26], as well as improve axial and rotational stability [9, 27]. Furthermore, a recent indirect meta-analysis [30] including both comparative and non-comparative studies on collared versus collarless stems for THA implanted by direct anterior approach, found that collared stems had significantly lower risk of complications, and tended to have lower risk of revisions. The authors of the present study believe that the collar can provide a protective effect against subsidence, for this reason both the conventional and shortened stems implanted were collared. Nonetheless, a potential drawback of collared stems may appear during revision surgery, as a well-fixed collared stem could be more challenging to revise than a well-fixed collarless stem.

This retrospective study has a number of limitations. First, the series represents a transition period, during which the surgeon started using a shortened stem for the first time; therefore, the outcomes would be expected to improve throughout the learning curve. Second, the only clinical score collected was the mHHS, which has been shown to have a ceiling effect [40]. Furthermore, there was a significant difference in preoperative mHHS between the groups, although the reason for this is not understood, as patients were not allocated in any specific way to the groups. Third, operating time was not evaluated in the present study; however, with the direct anterior approach, the surgeon is able to broach the femoral canal and insert the stem more easily when using the shortened compared to conventional stem. Fourth, a follow-up period of 60 days was chosen to study early clinical and radiographic outcomes because the surgeon performs a routine consultation at this time to evaluate each patient; however, we cannot infer if differences in outcomes across groups will exist in the mid- or long-term. Other studies in the literature have also selected a 60-day follow-up period to evaluate early outcomes [25, 35]; furthermore, complications, such as periprosthetic fractures and infections are known to occur in the first few weeks following THA [2]. A future study is currently underway to compare complication rates, radiographic outcomes, and clinical outcomes of primary THA with conventional versus shortened stems, at a minimum follow-up of 5 years.

### Conclusions

There were no significant differences between conventional-length and shortened stems in terms of postoperative complication rates, radiographic outcomes, and postoperative mHHS. However, patients implanted with shortened stems had less intraoperative complications, but lower net improvement in mHHS.

### Supplementary Information

**Additional file 1.**

## Data Availability

The datasets used and/or analysed during the current study are available from the corresponding author on reasonable request.
