# Supplementary figures and images for: No significant differences in 60-day postoperative complication rates between conventional and shortened stems
Source: J Exp Orthop. 2023 Dec 28;10:149. doi: 10.1186/s40634-023-00696-8 (PMC10754806; doi:10.1186/s40634-023-00696-8)

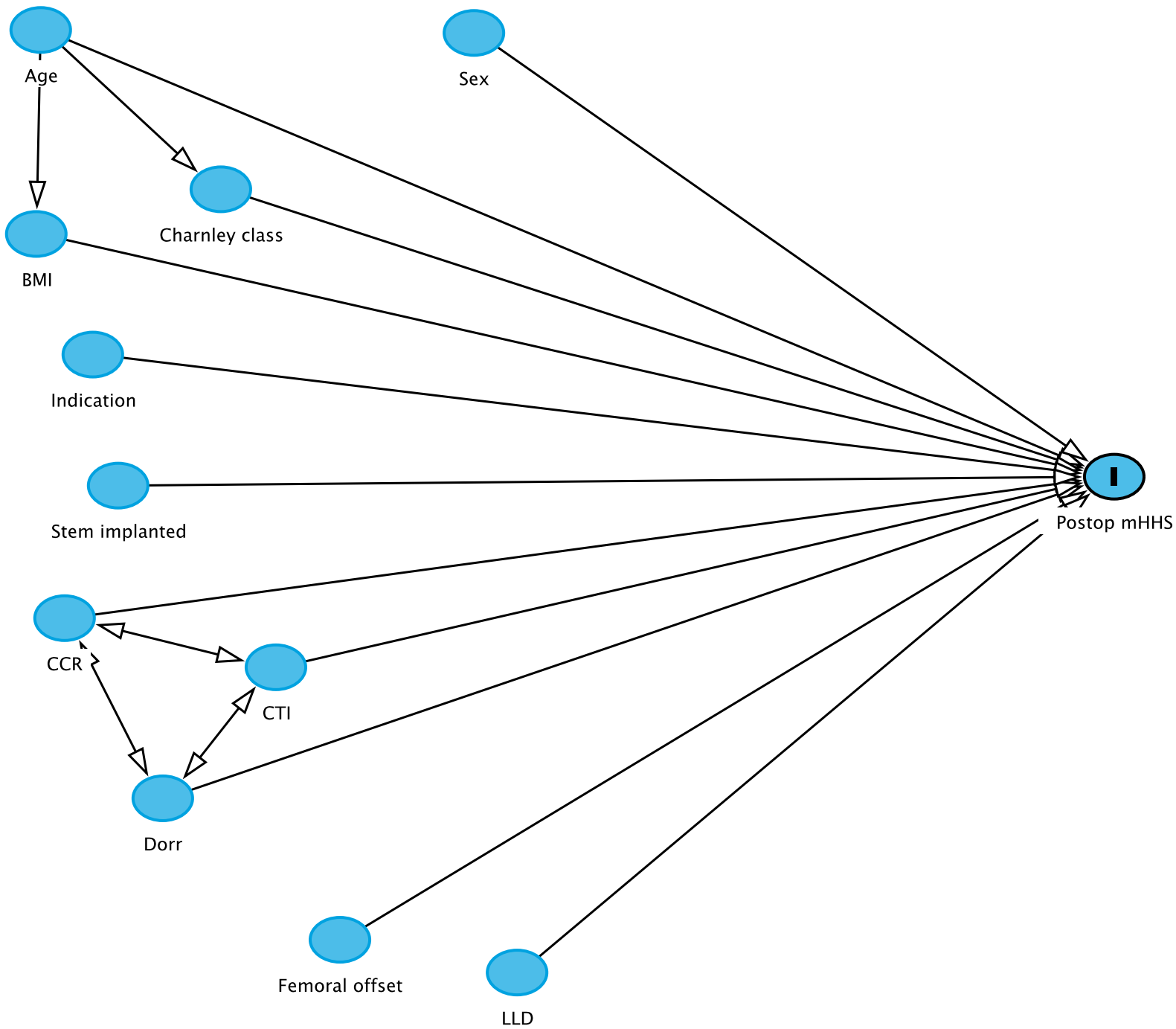

Supplement: Supplementary file 1 — Additional file 1. [file 40634_2023_696_MOESM1_ESM.pdf]
